# Screen exposure and social cognition: examining the relationships between screen time, smartphone addiction, internet addiction, and theory of mind components in adolescents

**DOI:** 10.1186/s41155-026-00381-6

**Published:** 2026-02-17

**Authors:** Didem Sain, Hasan Demirci, Yıldız Bilge

**Affiliations:** Department of Psychology, University of Health Sciences, Istanbul, 34668 Turkey

**Keywords:** Addiction, Adolescents, Screen exposure, Social cognition, Theory of mind

## Abstract

**Aim:**

Although there are a limited number of studies investigating the relationship between smartphones, internet addiction, and theory of mind (ToM) in adolescents, the effect of screen exposure on ToM is not well understood. This study aims to examine whether the components of ToM in adolescents are affected by screen exposure.

**Methods:**

The study sample comprised 293 students (164 females and 129 males) aged between 14 and 17 years. Data collection instruments included a Sociodemographic Data Form and an 11-item screen exposure instrument designed by the researchers. Additionally, the students were administered valid and reliable Turkish adaptations of the Smartphone Addiction Scale-Short Version (SAS-SV), the Young Internet Addiction Test-Short Form (YIAT-SF), the Reading the Mind in the Eyes Test (RMET), and the Dokuz-Eylül Theory of Mind Scale (DEToMS). Data were analyzed using the IBM SPSS Statistics 22.0 software package. The statistical analyses employed included independent samples t-tests, one-way analysis of variance (ANOVA), Pearson product-moment correlation coefficients, and mediation analysis using Model 4 of the PROCESS macro.

**Results:**

A significant but weak negative relationships was found between screen exposure and ToM components (for RMET *r =* -0.14, for DEToMS *r =* -0.19). There was a significant negative relationship between smartphone addiction and internet addiction and the ability to read minds from eyes (*r =* -0.017 and *r =* -0.015, respectively), but no significant relationship was found with other ToM skills. Screen time was found to mediate the relationship between social cognition and both smartphone and internet addiction. However, the indirect effect of screen time on smartphone addiction (18%) was found to be stronger than its effect on internet addiction (10%).

**Conclusion:**

These findings suggest that excessive screen exposure has a detrimental impact on social cognition. The observed association between smartphone and internet addiction and deficits in emotion recognition underscores the importance of interventions to enhance adolescents’ social interactions and mitigate the negative impact on ToM abilities. In this context, structured strategies, such as limiting screen time and implementing social skills training to improve emotion recognition, may be considered feasible interventions for educators and mental health professionals.

## Introduction

Screen exposure refers to the time an individual spends during the day interacting with visual screen-based electronic devices such as smartphones, tablets/iPads, computers/laptops, televisions, and gaming devices (Lewin et al., 2023). Using social media or communicating via text messaging also counts as screen exposure. Auditory activities such as talking on the phone and listening to music are not included in this time (Oswald et al., 2020). Today, children and adolescents live in a world equipped with electronic devices. Screen-based devices such as televisions, phones, tablets, and computers have become central to the daily lives of children and adolescents due to their entertainment value, interaction opportunities, and ease of access (Liu et al., 2022). As children and teenagers explore various content in digital spaces, their screen time rises significantly. These children, born into a rapidly evolving digital world, are often referred to as ‘digital natives’ or the ‘touchscreen generation.’ (Muppalla et al., 2023).

Electronic devices are increasingly being used in the field of education, and these devices have positive effects on students’ education and learning (Hale & Guan, 2015). It is suggested that using devices such as computers and tablets for purposes such as homework supports cognitive development and can increase academic success (Babic et al., 2017; Sanders et al., 2019). At the same time, virtual environments or social media accessed through these devices offer adolescents the opportunity to establish new relationships and develop existing relationships (Uhls et al., 2017).

Although electronic devices have beneficial effects on learning, accessing information, and communication, excessive screen exposure can have negative effects on physical, emotional/behavioral, and cognitive domains (Liu et al., 2022; Riesch et al., 2019). Many studies found that excessive screen time could cause various health problems in adolescents, such as musculoskeletal pain, eye strain, sleep problems (Calamaro et al., 2009; Falbe et al., 2015; Kim et al., 2017; Ulug et al., 2023), obesity (Nightingale et al., 2017; Robinson et al., 2017), type 2 diabetes risk (Nightingale et al., 2017), and metabolic syndrome (Kang et al., 2010). However, screen exposure in adolescents has also been shown to be associated with emotional/behavioral problems such as loneliness, difficulty making friends (Kim et al., 2009; Twenge & Campbell, 2018), depressive symptoms (Boers et al., 2019; Kremer et al., 2014), anxiety (Maras et al., 2015; Santiago et al., 2022), behavior problems (Song et al., 2020), tic disorders (Mohamed et al., 2025), hyperactivity, and inattention (Lissak, 2018; Wallace et al., 2023). A recent systematic review of 50 articles also found a negative association between screen time and mental well-being in adolescents (Santos et al., 2023).

Although it is suggested that the correct use of technological devices has positive effects on academic performance, some studies have shown that increased screen time is associated with lower academic performance (Cain et al., 2016; Hale & Guan, 2015; Peiró-Velert et al., 2014). Excessive screen time exposure has been shown to cause impairments in attention (Poujol et al., 2022; Santos et al., 2022), working memory, and executive function (Baumgartner et al., 2014). Children and adolescents are the most vulnerable group because they do not have adequate developmental skills to control screen exposure. Physical, cognitive, and behavioral problems that may develop due to excessive screen exposure can occur in this age group and may become chronic over time (Lissak, 2018; Liu et al., 2022).

Evidence indicates that studies on the effects of screen exposure in children and adolescents yield conflicting findings, demonstrating both beneficial and adverse outcomes. While the benefits of screen-based tools for education and social interaction have been highlighted (Babic et al., 2017; Hale & Guan, 2015; Sanders et al., 2019; Uhls et al., 2017), systematic reviews and meta-analyses show that increased screen time is associated with attention problems, executive function disorders, social skill deficits, and decreased mental well-being (Lissak, 2018; Santos et al., 2023). These discrepancies demonstrate that the effects of screen exposure are related to the duration and context of use, the type of content, and individual differences. However, the potential negative impacts on social-cognitive development and emotional health are critical for the long-term development of children and adolescents. Excessive or uncontrolled screen use, in particular, can negatively impact face-to-face interactions, family bonds, and the acquisition of basic social skills; therefore, focusing on the risks in this area can be important from both theoretical and practical perspectives.

Social cognition refers to the psychological processes involved in perceiving, encoding, storing, remembering, and organizing information necessary for effective social communication (Green et al., 2008). When social stimuli trigger a behavior, social cognition comes into play, guiding both automatic and voluntary behaviors (Adolphs, 2001). Social cognitive processes are divided into four different subgroups that are interrelated: emotion recognition, social perception, attribution style, and theory of mind (ToM) (Janssen et al., 2022). According to Brüne, the concept that best predicts social cognition among these subgroups is ToM (Brüne & Brüne-Cohrs, 2006). ToM is an individual’s ability to make accurate inferences from other people’s mental states, such as their thoughts, desires, beliefs, and intentions (Dziobek et al., 2005).

Vygotsky’s sociocultural theory argues that cognitive development is internalized through social interaction and cultural tools (Vygotsky, 1978). According to Vygotsky, the origin of complex mental processes lies in social interactions; in other words, cognitive development begins within a social context. These processes can be shaped by interactions with adults and through social interactions that individuals have with their peer groups. In this context, social interaction, language, and other cognitive tools enable the maturation of an individual’s social cognitive skills. Furthermore, neuroimaging studies have revealed that the medial prefrontal cortex, left temporopolar cortex, superior temporal sulcus, and anterior and posterior cingulate cortex are important neuroanatomical structures for ToM (Abu-Akel, 2003; Fletcher, 1995; Rilling et al., 2004; Vogeley et al., 2001). These brain regions associated with social cognitive processes continue to develop structurally and functionally during adolescence (Blakemore, 2008). Therefore, adolescence is a critical turning point for the maturation of ToM skills, both in terms of social interaction and neurobiologic maturation (Meinhardt-Injac et al., 2020; Blakemore, 2006).

ToM is crucial for successful social interaction because it allows us to predict and interpret the behavior of others based on their mental states (Doherty, 2008). It also aids social functioning by assisting individuals in coordinating their relationships with others (Watson et al., 1999). In this context, impairments in ToM negatively affect the individual’s ability to establish and maintain relationships with others (Kynast et al., 2021). For example, children with weak ToM cannot easily understand the difference between mistakes, lies, and deception, and this can cause children to have problems in social areas (Perner et al., 1994). Studies have shown that ToM ability in older children is associated with peer acceptance and may play an important role in the development of healthy peer relationships (Slaughter et al., 2002). This study was conducted with the consideration that prolonged screen exposure and various digital addictions were risk factors for the development of social cognition, aiming to highlight that increased screen time and digital addictions lead to fewer social interactions among children and adolescents. Consequently, they lack the necessary experiences for developing social cognition, which negatively impacts the development of these skills.

Research on how screen exposure affects adolescents’ cognitive, emotional, and behavioral development is still evolving in the literature. There are a limited number of studies investigating the relationship between the level of smartphone and internet addiction and ToM in adolescents (Aydın et al., 2020; Saatçioğlu et al., 2022). These studies also appear to focus on specific ToM tasks or are conducted with small sample sizes. However, the impact of screen exposure on all components of ToM is not well known. The aim of this study was to comprehensively examine whether all ToM components such as reading the mind in the eyes, first-degree false beliefs, second-degree false beliefs, irony comprehension, metaphor comprehension, empathic understanding, and faux pas comprehension were affected by screen exposure in adolescents. Also, to expand upon previous studies by determining the relationships between the level of smartphone and internet addiction and ToM components. In this context, the current study can offer important inferences that enable the evaluation of the effect of screen exposure on ToM, and the conceptualization of the effects of screen time on adolescents.

## Method

### Participants and procedures

This study employed a quantitative, cross-sectional research design utilizing a correlational survey method. The study sample comprised 293 adolescents aged 14 to 17 years (M = 15.42, SD = 0.94). To determine the appropriate sample size, an a priori power analysis was conducted using the G*Power software. With parameters set at α = 0.05, 1-β(Power) = 0.85, and a medium effect size of 0.15, the minimum required sample size was estimated at approximately 105. The final sample of 293 participants significantly exceeded this threshold, ensuring robust statistical power. Regarding sex distribution, 56% of the participants were female (n = 164) and 44% were male (*n =* 129). The age distribution was as follows: 14 years (*n =* 49, 16.7%), 15 years (*n =* 115, 39.3%), 16 years (*n =* 85, 29%), and 17 years (*n =* 44, 15%). Detailed demographic characteristics of the participants are presented in Table 1.

**Table 1:** Participants’ information on sociodemographic and screen exposure variables

Variables	Groups	(n)	(%)
Sex	female	164	56
	male	129	44
Age	14	49	16.7
	15	115	39.3
	16	85	29
	17	44	15
Mother’s education status	primary school	78	26.6
	middle school	42	14.3
	high school	76	26.2
	university	94	32.4
Father’s education status	primary school	39	23.5
	middle school	32	11.1
	high school	100	34.7
	university	119	40.6
Type of screen used	telephone	292	99.6
	computer	139	47.4
	tablet	34	11.6
	television	139	47.4
Screen intended use	homework	228	77.8
	social media	267	91.1
	playing games	185	63.1
	watching cartoons	66	22.5
	research	172	58.7
	other purposes	57	19.5
Screen exposure time	30 min—3 h	50	17,1
	3 h—6 h	145	49,5
	6 h—9 h	62	21,1
	9 h—12 h	28	9,6
	12 h and more	8	2,7

The inclusion criteria for the study were determined as being in the 14–17 years age group, volunteering to participate in the study, and not having a visual or hearing impairment. The exclusion criteria for the study were being aged under 14 and over 17 years, and not volunteering for the study for any reason. Data from participants who answered questions randomly and left some questions unanswered were excluded from the evaluation. A sociodemographic data form, the Screen Exposure Questionnaire, Smartphone Addiction Scale-Short Version (SAS-SV), the Young Internet Addiction Test-Short Form (YIAT-SF), Reading the Mind in the Eyes Test (RMET), and the Dokuz-Eylül Theory of Mind Scale (DEToMS) were administered to the participants. Before starting the study, a pilot study was conducted on a sample group of 30 people. The pilot study was conducted to determine the comprehension of the test and scale instructions, the completion time of the application, and possible problems. The application of the tests and scales took approximately 1 h. Before the application, the participants were presented with an informed consent form, which included information about the study, the confidentiality of the study, the voluntariness of participation, and the ability to withdraw from the study at any time without stating any reason. All data obtained from participants were stored in encrypted digital environments accessible only to the research team and were anonymized by removing all personally identifiable information prior to analysis. All stages of the research were conducted in compliance with relevant local data protection regulations and ethical principles concerning the protection of personal data. Approval for the research was obtained from the University of Health Sciences, Scientific Research Ethics Committee (311/2023).

### Assessments tools

#### Sociodemographic data form

This form was prepared by the researchers to obtain information about the demographic characteristics of the participants. It included questions about the participants’ age, sex, and education, as well as various subjects such as their parents’ education, occupation, family economic level, and number of siblings.

#### Screen exposure instrument

One of the most commonly used methods to assess screen time in children and adolescents is quantifying the daily time spent on computers/tablets, television, and, video games (Lucena et al., 2015). For this study, an 11-item form was developed to collect detailed information regarding adolescents’ screen exposure. Participants were asked about their daily exposure to various screen types (e.g., television, computer, phone) and the average time spent on these devices. Although no formal validity and reliability study was conducted, this instrument was designed with the sole purpose of determining the total screen time based on self-reports. Consequently, screen time was assessed by calculating the average daily duration (hours/minutes), and the responses were classified into five categories: 30 min–3 h, 3–6 h, 6–9 h, 9–12 h, and 12 h or more. Total screen time included both educational and non-educational screen use.

#### Smartphone Addiction Scale-Short Version (SAS-SV)

SAS-SV was developed by Kwon et al. to assess the risk of smartphone addiction (Kwon et al., 2013). The scale consists of 10 items, each scored between 1 and 6. The total score varies between 10 and 60. As the score on the scale increases, it is considered that the risk for smartphone addiction increases. The Turkish validity and reliability study of the scale was performed by Noyan et al. Cronbach’s alpha value was found as 0.90, and the cut-off point was 29.5 for both sexes (Şata & Karip, 2018).

#### Young Internet Addiction Test-Short Form (YIAT-SF)

The YIAT-SF, developed by Young, was converted into a short form by Pawlikowski et al. (Pawlikowski et al., 2013). The scale consists of 12 items, each scored from 1 to 5. The total score varies between 12 and 60. High scores on the scale indicate that the participant has a high level of internet addiction. The validity and reliability study of the test in Turkish was conducted by Kutlu et al. with university students and adolescents. The Cronbach alpha value of the scale was found as 0.86 (Kutlu et al., 2016).

#### Reading the mind in the eyes test (RMET)

The scale developed by Baron-Cohen et al. assesses a person’s ability to infer mental processes and emotions by looking at the expression in the eyes and surroundings of the person opposite them. Participants are shown 28 black-and-white pictures in the scale (Baron‐Cohen et al., 2001). After the participants are shown the pictures, they are asked to choose the option that best describes the mental state of the person in the picture, and mark it on the answer sheet. The test is considered a good indicator of emotion recognition and ToM skills because the options on the answer sheet are structured on complex emotions and intentions. The test is scored by giving one point for each correct answer. The possible score for the test varies between 0 and 28, with higher scores indicating better emotion reading, social cognition abilities, and ToM skills. The Turkish validity and reliability study of the scale was conducted on children and adolescents age over 6 years, and the Cronbach alpha value was found as 0.72 (Girli, 2014).

#### Dokuz-Eylül theory of mind scale (DEToMS)

Developed to assess ToM, one of the social cognition skills, DEToMS consists of seven stories and three picture tasks. In the story tasks, the individual is expected to listen to the stories carefully, and answer questions about them. Five of the story tasks require only one specific ability (first- and second-degree false beliefs, irony, metaphors, and faux pas), whereas the remaining two stories assess various aspects of ToM (empathy, irony, first- and second-degree false beliefs, and metaphors) through different questions within the same story. When scoring, 1 point is awarded for each correct answer given according to the answer key, and 0 points are awarded for each wrong answer. The score that can be obtained from story tasks varies between 0 and 15 points. In picture tasks, there are three pictures that make up a story, and the fourth picture needs to be chosen from among the two pictures in accordance with the question asked about the story. One of these pictures evaluates first-degree false beliefs, one evaluates second-degree false beliefs, and one evaluates empathy. If the correct picture is selected, 1 point is given, if the wrong picture is selected, 0 points are given, and the picture tasks can be scored between 0 and 3 points. The range of total scores that can be obtained from the entire scale is 0–18 points. The application takes approximately 15–20 min. The Turkish validity and reliability study of the scale was conducted by Değirmencioğlu (Değirmencioğlu, 2008).

### Statistical analysis

Data analyses were performed using the IBM SPSS Statistics v. 25 software package. Data were initially collected from 310 students; however, 13 participants were excluded due to missing data or random responding patterns. Additionally, four participants identified as multivariate outliers were removed based on Mahalanobis distance values. Consequently, the final analyses were conducted with the remaining 293 participants. Following data cleaning, skewness and kurtosis values were calculated to assess the normality of the scales, along with means and standard deviations. Descriptive analyses were performed using Pearson’s product-moment correlation analysis for the relationships between continuous variables. To test the hypothesized model, mediation analysis was conducted using Model 4 of Hayes’ PROCESS macro (Hayes, 2017). The significance level was set at.05 for all analyses.

## Results

### Internet usage characteristics and examination of variables according to demographic characteristics

Some 99.6% of the participating students stated that they were exposed to screens using phones, and 47.4% using computers. Fifty (17.1%) of the participants reported being exposed to screens for 30 min to 3 h per day, 49.5% (*n =* 145) for 3–6 h per day, 21.1% (*n =* 62) for 6–9 h per day, 9.6% (*n =* 28) for 9–12 h per day, and 2.7% (*n =* 8) for more than 12 h per day. Students reported that they used screens for doing homework, 91.1% (*n =* 267) for social media, 63.3% (*n =* 185) for playing games, 22.5% (*n =* 66) for watching cartoons, 58.7% (*n =* 172) for research, and 19.5% (*n =* 57) for other purposes (Table 1).

Skewness and kurtosis coefficients were examined to assess the normality of the data distribution. The skewness and kurtosis values for the continuous variables in the study were found to range between + 2.5 and −2.5. The data were assumed to be normally distributed because these values fall within the acceptable limits for normal distribution suggested in the literature (Kline, 2011). When comparing variables by sex, girls were found to have significantly higher scores on the SAS, DEToMS, and SE than boys (Table 2). Although no significant differences were found among the variables based on income, comparisons among age groups revealed that 14-year-olds (M = 31.42, SD = 8.29) scored significantly higher on internet addiction than 17-year-olds (M = 26.16, SD = 7.26) (F = 3.72, p = 0.012, *p <* 0.05). Additionally, the 14-year-old group (M = 14.10, SD = 2.55) was observed to have higher scores on the DEToMS compared with the 16-year-old (M = 12.75, SD = 2.29) and 17-year-old (M = 12.77, SD = 2.12) groups (F = 3.77, p = 0.011, *p <* 0.05).

**Table 2:** Continuous variables according to gender

	**Girls (M)**	**Girls (SD)**	**Boys (M)**	**Boys (SD)**	**t**	**p**
Smart Addiction	27.91	9.29	25.10	8.62	2.65	.008^**^
Internet Addiction	29.08	8.02	27.60	7.76	1.60	.111
Reading the Mind in the Eyes	20.41	2.56	20.21	2.63	.64	.522
Dokuz-Eylül Theory of Mind	13.61	2.33	12.72	2.61	0.10	.002^**^
Screen Time	14.71	8.05	17.05	8.60	−2.40	.017^**^

*df* = 291,*p< .05, **p< .01

### Results of correlation analysis between variables

Pearson’s correlation coefficients and effect sizes based on Cohen’s (1988) criteria were calculated to examine the relationships between age, screen time, smartphone addiction, internet addiction, and reading mind from eyes scores. According to the analysis results, no significant relationships were found between age, screen time, and RMET scores. However, a significant negative correlation was found between age and SAS-SV (*r =* −0.12, *p <* 0.05, small effect), YIAT-SF (*r =* −0.16, *p <* 0.01, small effect), and DEToMS scores (*r =* −0.19, *p <* 0.01, small effect). There was a statistically significant positive relationship between screen time and SAS-SV (*r =* 0.40, *p <* 0.01, medium effect) and YIAT-SF scores (*r =* 0.26, *p <* 0.01, small effect). There was a significant negative correlation between screen time and RMET (*r =* −0.14, *p <* 0.05, small effect) and DEToMS scores (*r =* −0.19, *p <* 0.01, small effect). Also, a statistically significant positive relationship was found between the scores obtained from SAS-SV and YIAT-SF (*r =* 0.65, *p <* 0.01, large effect). No significant relationship was found between the scores obtained from SAS-SV and DEToMS, but a significant negative relationship was found between the RMET scores (*r =* −0.16, *p <* 0.01, small effect). No significant relationship was observed between the YIAT-SF and DEToMS scores; however, there was a significant negative relationship with RMET (*r =* −0.14, *p <* 0.05, small effect). Correlation results are presented in Table 3.

**Table 3:** Correlation analysis results between variables

	**M ± SD**	**SK/CU**	**2**	**3**	**4**	**5**	**6**
1	15.42 ± 0.94		-.07	-.12^*^	-.16^**^	.10	-.19^**^
2	5.94 ± 2.84	1.07/1.35	1	.40^**^	.26^**^	-.14^*^	-.19^**^
3	26.68 ± 9.10	.39/-.23		1	.65^**^	-.17^**^	.06
4	28.43 ± 7.93	.78/.99			1	-.15^*^	.02
5	20.32 ± 2.58	-.55/.89				1	.18^**^
6	13.22 ± 2.49	-.90/1.83					1

1. Age, 2. Screen Time, 3. Smartphone Addiction Scale, 4. Young Internet Addiction, 5. Reading the Mind in the Eyes, 6. Dokuz-Eylül Theory of Mind

^*^p < .05, **p < *.01*

### Mediation analysis

**Fig. 1:**
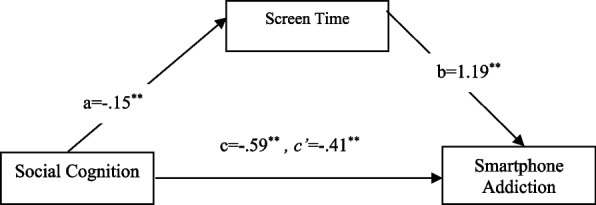
Mediating effect of screen time on the relationship between social cognition and smartphone addiction

Mediation analysis was performed using Model 4, which evaluates simple mediation, using Hayes’ (2017) Process macro plugin. In both models, RMET scores were used as the independent variable for social cognition. The direct effect of social cognition scores, the independent variable in the first model created, on screen time, was found valid (a) (B = −0.15, SH = 0.06, t = −2.36, 95% CI: [−0.2760 to −0.0253]; *p <* 0.05). The direct effect of screen time on smartphone addiction scores, the dependent variable of the model, was significant (b) (B = 1.19, SH = 0.17, t = 6.87, 95% CI: [0.8528–1.5370]; *p <* 0.001). The total effect of social cognition, the independent variable, on smartphone addiction (c) (B = −0.59, SE = 0.20, t = −2.92, 95% CI: [−0.9936 to −0.1931]; *p <* 0.01), and its direct effect (c’) were found to be significant (B = −0.41, SE = 0.19, t = −2.17, 95% CI [−0.7887 to −0.0380]; *p <* 0.05). The bootstrap method was used to test whether the effect of the mediating variable was significant, and the fact that the confidence intervals did not include zero indicates significant mediating relationships. The results indicated that the indirect effect on smartphone addiction scores was significant at 18% (B = −0.18, SH = 0.09, 95% CI: [−0.3693 to −0.0132]) (Fig. 1). Additionally, the entire model, which explains 3% of the variance, was found to be significant (F(1,291) = 8.51, *p <* 0.05).

**Fig. 2:**
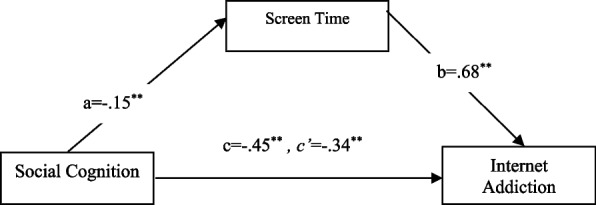
Mediating effect of screen time on the relationship between social cognition and internet addiction

The direct effect of social cognition scores, which is the independent variable in the second model, on screen time was significant (a) (B = −0.15, SH = 0.06, t = −2.36, 95% CI: [−0.2760 to −0.0253]; *p <* 0.05). The direct effect of screen time on internet addiction scores, the dependent variable of the model, was found valid (b) (B = 0.68, SH = 0.16, t = 4.27, 95% CI: [0.3667–0.9926]; *p <* 0.001). The total effect of social cognition, the independent variable, on internet addiction (c) (B = −0.45, SH = 0.18, t = −2.50, 95% CI: [−0.7946 to -.0945]; *p <* 0.05), and its direct effect (c’) were found to be significant (B = −0.34, SH = 0.17, t = −1.96, 95% CI: [−0.6855 to −0.0001]; *p <* 0.05). The bootstrap method was used to test whether the effect of the mediating variable was significant, and the fact that the confidence intervals did not include zero indicated significant mediating relationships. The results indicated that the indirect effect on internet addiction scores at a 10% level was significant (B = −0.10, SH = 0.06, 95% CI: [−0.2290 to −0.0066]) (Fig. 2). Additionally, the entire model, which explained 2% of the variance, was found to be significant (F(1,291) = 6.25, *p <* 0.05).

## Discussion

This study examined the relationship between ToM, and the duration of exposure to visual screen-based electronic devices such as smartphones, tablets/iPads, computers, and televisions in adolescents. Among the main results of the present study, the following can be highlighted; (a) As screen exposure time increases, ToM skills—especially reading the mind in the eyes, first-degree false beliefs, second-degree false beliefs, understanding irony, understanding metaphors, empathic understanding, and recognizing faux pas—decline, (b) There is a negative correlation between smartphone addiction and internet addiction levels and the ability to read mind from eyes, (c) There is no relationship between smartphone addiction and internet addiction, and other ToM skills, (d) The relationship between social cognition and smartphone and internet addiction increases with the mediating effect of screen time.

The main finding of the current study is that as screen exposure increases in adolescents, ToM skills decrease. To our knowledge, there is no study in the literature that evaluates the relationship between screen exposure and ToM in adolescents. Several studies with preschool children have focused on television exposure and performance on ToM tasks. Nathanson et al. (2013) reported that preschool children with a television in their bedroom and high levels of background exposure exhibited diminished abilities to accurately infer the mental states of others, including their thoughts, beliefs, desires, and intentions. Another study found a relationship between television exposure and poor ToM skills in children aged 3–6 years (Nathanson & Fries, 2014). Since ToM skills in the preschool period are more dependent on environmental inputs and face-to-face interactions, television exposure is expected to have a more direct impact on these skills. Our findings support these findings that screen exposure negatively affects ToM skills in children and extend this effect into adolescence. Although ToM is more established and cognitively developed during adolescence, high screen exposure can negatively impact social-cognitive processes, particularly through passive use and reduced face-to-face social interaction.

Another finding of the current study is that as the level of internet and smartphone addiction increases in adolescents, the performance of reading minds from eyes decreases. However, no relationship was found between either type of addiction and other ToM skills. These findings suggest that heavy engagement with the internet and smartphones affects the capacity to interpret socioemotional cues from other individuals, but does not affect ToM skills sas detailed above. The possible reason RMET is selectively affected is that this task requires visual-emotional processing, while DEToMS tests more of the cognitive components of ToM. Studies conducted with adolescent samples in the literature have consistently documented that internet addiction negatively affects the performance of reading mind from eyes. Akdeniz et al. found a negative relationship between internet addiction and the ability to read mind from eyes in their studies of adolescents aged 14–16 years (Akdeniz et al., 2020). It has been found that adolescents with internet addiction have a weaker ability to recognize facial expressions (Ge et al., 2017), and have deficiencies in both social cognition and emotion regulation skills (Saatçioğlu et al., 2022). Similar results were obtained in studies conducted on university students. A negative relationship was found between internet addiction and RMET among university students (Shiri et al., 2024). In another study, university students with pathological internet use had lower ToM scores than those without (Korkmaz et al., 2018). A study conducted by Lee et al. found a negative relationship between excessive smartphone use and the ability to read mind from eyes in university students (Lee et al., 2022). These findings obtained in our study between internet addiction and the ability to read mind from eyes were consistent with other studies in the literature.

In both models created for mediation analysis, it was determined that the relationship between ToM and smartphone and internet addiction increased with the mediation effect of screen time. The significant mediating role of screen time in both models supports that screen time is a critical intermediary mechanism in the relationship between ToM and addiction. The results show that screen time is both a passive measure of use and an active element that fosters addictive behaviors. The basis of interacting with people is the ability to recognize their emotions and cognitive processes and make inferences about how they will behave (Singer, 2006; Völlm et al., 2006). It is necessary to have advanced ToM skills to be able to make accurate inferences from the mental states of others, such as their intentions and beliefs. High screen exposure may play a role in adolescents’ inadequate development of ToM skills, leading to difficulties in social relationships and avoidance of social interactions. Adolescents may seek to alleviate these challenges by increasing their screen time, potentially resulting in a detrimental cycle. Elevated screen exposure can negatively impact social cognition, which in turn influences the quality and quantity of social relationships. However, it is known that excessive screen use in children and adolescents causes attention problems, working memory impairment, and executive function disorders (Baumgartner et al., 2014; Poujol et al., 2022; Santos et al., 2022). Screen exposure can lead to cognitive overload, negatively impacting information processing and attention allocation. This, combined with or independently of a lack of social interaction, can diminish the performance of ToM.

ToM ability is a basic skill that must be acquired during the transition to adolescence, playing a critical role in the individual’s identity development and ability to establish prosocial relationships (Bosacki et al., 2020). At the same time, factors such as conversations between parents and children and interactions with other people have an important effect on the acquisition and development of ToM (Nathanson & Fries, 2014; Rusli et al., 2021). Studies have shown that children who grow up in a language-rich home environment where mental states and emotions are frequently discussed perform better on ToM tasks than children who grow up in different environments (Adrián et al., 2007; de Rosnay & Hughes, 2006). In this context, screen exposure may eliminate opportunities for rich social interaction and face-to-face conversations that are essential for ToM development. It is known that in environments where mobile devices are used intensively, the quality of establishing closeness with others and conversation is negatively affected, which prevents meaningful interaction (Misra et al., 2016; Przybylski & Weinstein, 2013; Sigman, 2012). At the same time, intensive screen exposure negatively affects children’s social skill development by limiting their opportunities to gain social experience in real life (Sigman, 2012). It has been concluded that children’s ToM skills may be negatively affected as they spend more time watching television, which may reduce their communication with their parents and their opportunities to experience real relationships (Nathanson & Fries, 2014). As a result of intensive screen exposure, the decrease in shared activities that adolescents engage in with their families and friends, and the limited face-to-face interactions, especially at home, may cause individuals to fail to experience social cues sufficiently. Consequently, a potential explanation for the observed association between screen exposure and ToM among adolescents in this study may be the restrictive social interaction environment resulting from increased screen use.

Neuroimaging studies have revealed that the medial prefrontal cortex, left temporopolar cortex, superior temporal sulcus, and anterior and posterior cingulate cortex are important neuroanatomic structures for ToM (Abu-Akel, 2003; Fletcher, 1995; Rilling et al., 2004; Vogeley et al., 2001). It has also been suggested that connections between the amygdala and orbitofrontal cortex (OFC) structures are part of the ToM circuit (Abu-Akel, 2003). Imaging studies on adolescent samples have shown that internet and smartphone addiction leads to structural changes in many areas of the brain and is associated with functional abnormalities (Chun et al., 2018; Lin et al., 2012; Wee et al., 2014; Yuan et al., 2011). One of these studies revealed a decrease in cortical thickness in structures such as the left lateral OFC and insular cortex in adolescents addicted to online games (Yuan et al., 2013). Another study reported a decrease in the thickness of the OFC in adolescents with internet addiction (Hong et al., 2013). Long-term internet addiction has been shown to cause atrophy in the bilateral dorsolateral prefrontal cortex (DLPFC), OFC, left rostral anterior cingulate cortex, and gray and white matter in the supplementary motor area (Yuan et al., 2011). Another study showed reduced neural activation in the DLPFC and dorsal anterior cingulate cortex in participants who used smartphones excessively (Chun et al., 2017). It has also been suggested that excessive screen time may affect the development of the corpus callosum, a structure that facilitates communication between the brain hemispheres, leading to difficulties in integrating social and emotional information (Takeuchi et al., 2015). Screen exposure’s reduction of face-to-face interactions may lead to a lack of real-time social interaction and feedback, which play a critical role in learning social norms and behaviors, impairing the natural development of brain regions responsible for empathy, perspective-taking, and emotional regulation. These brain regions overlapping in both ToM skills and neuroanatomic structures associated with screen exposure, internet, and smartphone addiction may be another possible cause of the ToM impairments we found with screen exposure in adolescents.

### Limitations and future research directions

Although this study has important findings, it has some limitations that should be considered when interpreting the results. First, the cross-sectional design of the study creates limitations in revealing causal relationships. Another limitation is that the scales used are based on self-reporting by adolescents, which may have caused the participants to have different attitudes towards providing accurate information about themselves. It remains unclear whether the variables in the study can be generalized to objective measurements. The effects of screen exposure may vary depending on the cultural and contextual conditions in which individuals find themselves. Socioeconomic status and access to digital resources can shape the nature of screen use, thereby influencing the potential effects of this exposure in either positive or negative directions. Therefore, it is important to consider these contextual factors when interpreting the findings. It is recommended that future research uses longitudinal designs to better understand causal relationships. Longitudinal studies on adolescents could provide deeper insights into how screen exposure shapes development and identify critical time periods for intervention. Additionally, examining the effects of screen time across different age groups and socioeconomic backgrounds may contribute to filling existing gaps in the literature. Finally, studies combining screen exposure, ToM tasks, and neuroimaging techniques could make it possible to directly examine how screen exposure affects ToM skills.

## Conclusion

The present study found significant negative correlations between screen exposure time and scores on mind reading from the eyes as well as other ToM skills. Additionally, significant and negative correlations were found between smartphone and internet addiction and the ability to read mind from eyes. However, no significant relationship was found between the scores obtained from DEToMS, which includes other ToM skills, and smartphone and internet addiction. Screen exposure was examined in various dimensions and internet and smartphone addiction were also evaluated, and screen exposure was evaluated in a broader perspective. The literature shows that excessive screen exposure in children and adolescents has negative consequences in various areas, including motor development, cognitive development and academic achievement (Suggate & Martzog, 2021). The current study shows that excessive screen exposure also has negative effects on social cognition. Limiting screen exposure may be a good starting point to increase adolescents’ social interactions and reduce the negative effects of screen exposure on ToM. For example, some researchers recommend that adolescents spend at most 2 h a day on screen time (Strasburger et al., 2013). A recent study revealed a negative correlation between total screen time and, cognitive performance, showing that children with more than 3 h of screen exposure per day had significantly lower average cognitive scores compared to those with 2 h or less of screen exposure (Kaushik et al., 2025). In this context, activities that support physical and social interaction should be encouraged, and screen-free time should be integrated into daily routines. Additionally, structured family interaction programs and school-based digital detox initiatives can be implemented. Given the negative public health consequences associated with excessive screen media use, developing educational policies that integrate digital literacy into school curricula to create a safe and healthy digital environment for adolescents is critically important.

## Data Availability

The datasets used and analyzed in the current investigation are accessible from the corresponding author upon reasonable request.

## Abbreviations

DEToMS: Dokuz-Eylül Theory of Mind Scale
RMET: Reading the Mind in the Eyes Test
SAS-SV: Smartphone Addiction Scale-Short Version
ToM: Theory of Mind
YIAT-SF: the Young Internet Addiction Test-Short Form
